# Atmospheric Pressure Plates

**Published:** 1859-06

**Authors:** Horace T. Fogg

**Affiliations:** San Paulo, Brazil, S. A.


### ATMOSPHERIC PRESSURE PLATES.

To the Editors of the Dental News Letter—

*Gentlemen:*—In consequence of reading an article in the above journal, Vol. xi. No. 3, on a good method of abtaining a complete fit of atmospheric pressure plates, I have resolved to request you to publish a description of the manner in which I have made these plates for the last eight years, if you think it worthy of a place in your valuable periodical.

All depends upon a perfect impression of the gums, and for this purpose I have never found anything better than good, pure yellow beeswax, which I have ready prepared in thin sheets; warm it by the heat from a spirit-lamp, working it well with the fingers until it is uniformly smooth and soft throughout. On the wax impression I cut out with an ordinary pocket-knife, the space for the air-chamber, taking great care to preserve the edge around it perfect, giving it the depth necessary, but smooth it off afterward on the plaster-cast. When the wax impression comes from the mouth, it should be large enough to admit of the plaster-cast being cut in a round shape; if not, make it so by adding more wax on the outsides without altering its shape; then fill up with plaster of paris, an inch high is enough; when set, separate it from the wax and trim the mould so that it can afterward easily be released from the metal; finish off the air-chamber, running the point of a knife (or any sharp instrument) around the edge, making a small crease, and cut the mould round, so that a strip of paper can be folded around it sufficiently tight to hold the metal when poured in. But first oil this plaster-cast, and make another from it by pouring plaster over it; when set and trimmed around, separate it and dry it to keep for further use. Also dry the plaster-cast which came from the wax impression, and when perfectly free from moisture, wrap a strip of ordinary paper around it three inches in width, so that it forms a cup to receive the metal, and fasten it with a wafer; pour in upon this, melted fusible metal, (8 parts bismuth, 5 lead, and 3 of tin), when it is almost ready to cool, and in this way get a perfect female mould; when cold separate it from the plaster with care, cover it with lamp-smoke, wrap paper around it in the same manner, and pour in fusible metal for the male mould. Then make a second set of dies from the same plaster-cast, which, if properly prepared at first, will still be perfect; and still another set may be made, if thought necessary, although I find this number sufficient. While these are cooling, make a male and female die in lead from the same plaster of paris mould for one, and the one taken from that already put by for this use, for the other; wrapping paper around them also to hold the lead, but put enough to surround the plaster four or five times; pour in slowly the melted lead almost cool, and the paper will hold. You have then two (or more) sets of dies of fusible metal, and one set of lead. Of the fusible metal, select the most perfect to be used at last, and commence striking up the plate between a male fusible metal die and female lead. When the plate is getting into shape, use the female metal die with the male lead, and here is the advantage to be got from having a male lead die, which, being softer than the fusible metal, allows the latter to stamp up the plate more perfectly around the air-chamber; use these only once or twice, then use the same male and female fusible metal dies together, until the plate is as perfect as it possibly can be made with these, and shows every mark of the gums, also distinctly the edge around the airchamber. Now finish the plate with the new dies, first using the female fusible metal with the male lead, only once: and then the male fusible metal with the female lead, until the plate exactly fits the plaster-cast; striking it up once or twice will be sufficient, and you have a perfect atmospheric pressure plate as easily and as quickly made as an ordinary one, with the exception of the extra trouble and time in making the moulds, without the use of iron pans or anything else but a few strips of common paper and the pliers to turn over the edge of the plate in the commencement, besides using less metal. At least I have never known one to fail, either for a whole or a partial set; and sincerely hope some one will profit by my experience.

If there are a few defects in the female plaster-cast, as well as in the two lead dies, it does not matter; the cast becomes more perfect from use with the fusible metal; besides, they only assist in stamping up the plate, which is made perfect by the fusible metal dies.

I use fusible metal in preference to any other for all dies; but it must be allowed to cool gradually, and never be put into cold water for this purpose, which makes it very brittle.

Respectfully yours, etc., HORACE T. FOGG.

*San Paulo*, Brazil, S. A., Dec. 5, 1858.

